# Coenzyme Q_10_ Supplementation Improves Adipokine Levels and Alleviates Inflammation and Lipid Peroxidation in Conditions of Metabolic Syndrome: A Meta-Analysis of Randomized Controlled Trials

**DOI:** 10.3390/ijms21093247

**Published:** 2020-05-04

**Authors:** Phiwayinkosi V. Dludla, Patrick Orlando, Sonia Silvestri, Fabio Marcheggiani, Ilenia Cirilli, Tawanda M. Nyambuya, Vuyolwethu Mxinwa, Kabelo Mokgalaboni, Bongani B. Nkambule, Rabia Johnson, Sithandiwe E. Mazibuko-Mbeje, Christo J. F. Muller, Johan Louw, Luca Tiano

**Affiliations:** 1 Biomedical Research and Innovation Platform, South African Medical Research Council, Tygerberg 7505, South Africa; pdludla@mrc.ac.za (P.V.D.); rabia.johnson@mrc.ac.za (R.J.); christo.muller@mrc.ac.za (C.J.F.M.); johan.louw@mrc.ac.za (J.L.); 2 Department of Life and Environmental Sciences, Polytechnic University of Marche, 60131 Ancona, Italy; p.orlando@univpm.it (P.O.); s.silvestri@univpm.it (S.S.); f.marcheggiani@univpm.it (F.M.); ilenia.cirilli@unicam.it (I.C.); 3 School of Pharmacy, University of Camerino, 62032 Camerino, Italy; 4 School of Laboratory Medicine and Medical Sciences, College of Health Sciences, University of KwaZulu-Natal, Durban 4000, South Africa; mnyambuya@nust.na (T.M.N.); 218081787@stu.ukzn.ac.za (V.M.); 218086707@stu.ukzn.ac.za (K.M.); nkambuleb@ukzn.ac.za (B.B.N.); 5 Department of Health Sciences, Faculty of Health and Applied Sciences, Namibia University of Science and Technology, Windhoek 9000, Namibia; 6 Division of Medical Physiology, Faculty of Health Sciences, Stellenbosch University, Tygerberg 7505, South Africa; 7 Department of Biochemistry, Faculty of Natural and Agricultural Sciences, North-West University, Mmabatho 2745, South Africa; sithandiwe.mazibukombeje@gmail.com; 8 Department of Biochemistry and Microbiology, University of Zululand, KwaDlangezwa 3880, South Africa

**Keywords:** coenzyme Q_10_, ubiquinone, metabolic syndrome, metabolic complications, non-alcoholic fatty liver disease, hypertension, adipokines, inflammation, oxidative stress, lipid peroxidation

## Abstract

Evidence from randomized controlled trials (RCTs) suggests that coenzyme Q_10_ (CoQ_10_) can regulate adipokine levels to impact inflammation and oxidative stress in conditions of metabolic syndrome. Here, prominent electronic databases such as MEDLINE, Cochrane Library, and EMBASE were searched for eligible RCTs reporting on any correlation between adipokine levels and modulation of inflammation and oxidative stress in individuals with metabolic syndrome taking CoQ_10_. The risk of bias was assessed using the modified Black and Downs checklist, while the Grading of Recommendations Assessment, Development and Evaluation (GRADE) tool was used to evaluate the quality of evidence. Results from the current meta-analysis, involving 318 participants, showed that CoQ_10_ supplementation in individuals with metabolic syndrome increased adiponectin levels when compared to those on placebo (SMD: 1.44 [95% CI: −0.13, 3.00]; *I*^2^ = 96%, *p* < 0.00001). Moreover, CoQ_10_ supplementation significantly lowered inflammation markers in individuals with metabolic syndrome in comparison to those on placebo (SMD: −0.31 [95% CI: −0.54, −0.08]; *I*^2^ = 51%, *p* = 0.07). Such benefits with CoQ_10_ supplementation were related to its ameliorative effects on lipid peroxidation by reducing malondialdehyde levels, concomitant to improving glucose control and liver function. The overall findings suggest that optimal regulation of adipokine function is crucial for the beneficial effects of CoQ_10_ in improving metabolic health.

## 1. Introduction

Metabolic diseases are acknowledged to greatly affect the quality of life to those affected in addition to be the leading cause of death worldwide [1,2]. Overnutrition and sedentary lifestyle are currently known to be some of the major factors driving the rapid rise in metabolic diseases [3,4], in both developed and developing countries [5,6]. In fact, the past few decades have seen a drastic increase in the number of overweight and obese individuals [7]. Of major concern is that obesity, together with other metabolic complications such as hypertension, type 2 diabetes (T2D), and non-alcoholic fatty liver disease (NAFLD) can significantly accelerate the risk of heart failure, leading to reduced lifespan of the general population [4,8,9]. Consequently, there has been a great interest in understanding the pathophysiological mechanisms associated with the development of metabolic complications such as T2D and NAFLD [10]. This is especially important to identify molecular mechanisms for therapeutic target to alleviate metabolic complications.

Complex pathological mechanisms have thus far been linked with the development and aggravation of metabolic syndrome. For instance, a state of dyslipidemia, which is characterized by abnormally elevated serum levels of triglycerides and cholesterol, has been shown to promote enhanced ectopic lipid accumulation and subsequent injury to various body organs [4,11,12]. Increased organ and arterial lipid accumulation can induce insulin resistance and cause endothelial dysfunction, mostly through exacerbating inflammation and oxidative stress. Indeed, elevated pro-inflammatory cytokines such as C-reactive protein (CRP), interleukin (IL)-6, including lipid peroxidation makers, such as malondialdehyde (MDA) levels, have been identified in patients with dyslipidemia [13,14,15]. Consequently, our group and others are scrutinizing literature on the therapeutic value of various compounds in protecting against metabolic diseases, especially their impact on adipose tissue function and ameliorative properties on inflammation and oxidative stress [16,17,18,19].

Coenzyme Q_10_ (CoQ_10_), also referred to as ubiquinone, is attracting much interest due to its envisaged health benefits [20,21]. Interestingly, CoQ_10_ is present in all eukaryotic cells, and it forms a crucial part of the electron transport chain. Notably, its deficiency is associated with the generation of oxidative stress and myocardial damage in the diabetic state [22]. Whereas, dietary intake of CoQ_10_ is directly linked with enhanced levels of ubiquinol-10 within the human body, which is further crucial for blocking lipid peroxidation by protecting against low-density lipoprotein oxidation in conditions on metabolic syndrome [23,24]. Beyond its impact on lipid peroxidation, published evidence from randomized clinical trials (RCTs) shows that CoQ_10_ remains important in ameliorating inflammation and improving adipokine function in conditions of metabolic syndrome [25,26,27]. Although other meta-analysis of RCTs has been conducted on the effects of CoQ_10_ on markers of inflammation or oxidative stress [28,29,30], including assessing adiponectin levels [31], such information remains limited for being too specific and does not collectively give a better picture on the influence of this quinone on conditions of metabolic syndrome. Therefore, this meta-analysis assesses essential evidence on the modulatory effects of CoQ_10_ on adipokine function, including its impact on markers of inflammation and lipid peroxidation in individuals with metabolic syndrome.

## 2. Results

### 2.1. Selection and Characteristic Features of Included RCTs

**Figure 1:**
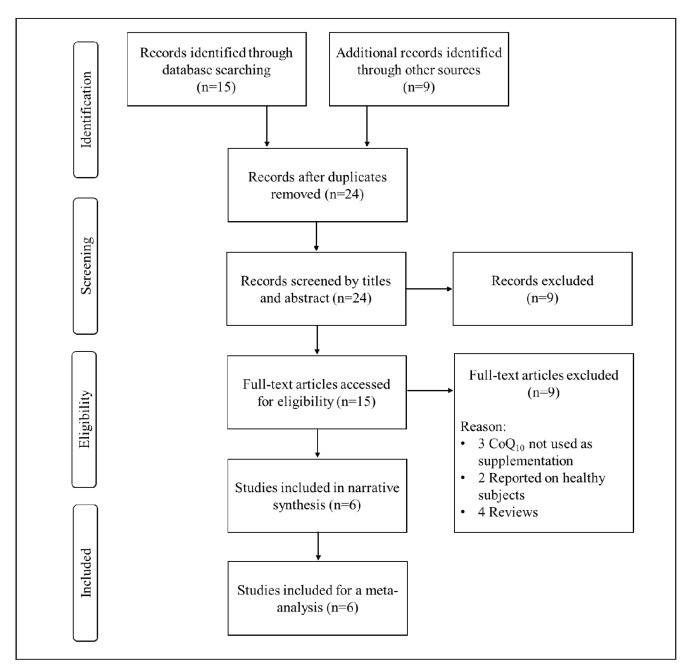
An overview of flow diagram of included studies.

Approximately, 24 RCTs were retrieved from relevant online databases reporting on the impact of CoQ_10_ on adipokine levels in individuals with metabolic syndrome. Subsequently, six RCTs met the inclusion criteria as displayed in Figure 1. Other studies were excluded for reasons such as being out of scope or not reporting on primary findings. The RCTs reporting on the impact of CoQ_10_ on adipokine levels in individuals without the metabolic syndrome were excluded from the meta-analysis [32].

The general characteristic features of the included studies are summarized in Table 1. In brief, all included RCTs were published in peer-reviewed journals, each year between 2014 and 2018. Briefly, this study was composed of a total of 318 participants with an average age of 48 years, and the majority of these being female (73%). The majority of participants included in the current meta-analysis were those with T2D (*n* = 176), NAFLD (*n* = 82), and hypertension (*n* = 60). The prominent doses of CoQ_10_ used were 100 or 200 mg/daily for a minimum of four weeks, up to three months (Table 1).

### 2.2. Risk of Bias and Quality of RCTs

The risk of bias and quality of six included studies was assessed by Kabelo Mokgalaboni (K.M.) and Vuyolwethu Mxinwa (V.M.), according to a modified Downs and Black’s guideline, as previously mentioned. The overall median score range of the included studies was 22 (19–24), with five of them rated excellent (21–24 scores) and one rated as good (19 scores). In addition, KM used the Cohen’s Kappa interrater to assess the degree of agreement in terms of quality assessment scores of the included studies [33]. The levels of agreement between the two reviewers (K.M. and V.M.) were scored as moderate to perfect. Furthermore, all included studies scored high in reporting bias 9 (9–10) out of ten possible scores (overall agreement 90%, Kappa = 0.89), external validity 2 (1–3) out of three possible scores (overall agreement 62%, Kappa = 0.52), internal validity 6 (5–6) out of seven possible scores, (overall agreement 79.6%, Kappa = 0.83) and selection bias with an average median scores of 5 (3–6) out of a six possible scores (overall agreement 80.95%, Kappa = 0.76).

### 2.3. Publication Bias

Publication bias was assessed using funnel plots for each measured outcome. Notably, visual inspection of funnel plots showed symmetry in all reported outcomes.

### 2.4. Data Synthesis

#### 2.4.1. CoQ_10_ Supplementation Improved Adipokine Levels

**Figure 2:**
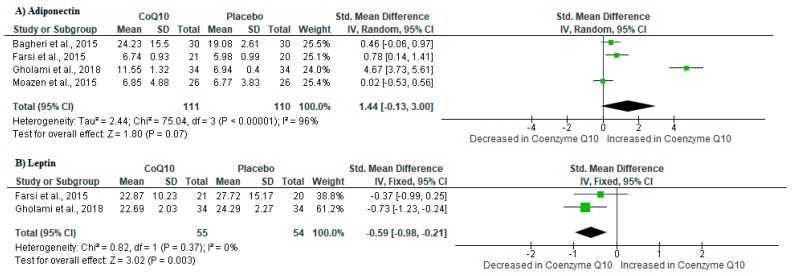
The impact of coenzyme Q_10_ (CoQ_10_) supplementation on adipokine levels in individuals with metabolic syndrome. Briefly, the results showed that CoQ_10_ supplementation increased the levels of adiponectin (**A**) whilst reducing that of leptin (**B**) in included randomized controlled trials.

It is now well-established that adipose tissue functions as a vital organ for the secretion of adipose-derived factors or rather adipokines which play a key role in the regulation of inflammation [37]. In fact, one of these adipokines is adiponectin, an anti-inflammatory metabolite that has been shown to improve insulin sensitivity [38,39]. Interestingly, pooled estimates showed that CoQ_10_ supplementation in individuals with metabolic syndrome increased adiponectin levels when compared to those on placebo (SMD: 1.44 [95% CI: −0.13, 3.00]; *I*^2^ = 96%, *p* < 0.00001) (Figure 2A). On the other hand, leptin has been long identified as a pro-inflammatory metabolite that exacerbates inflammation in conditions of metabolic syndrome [40]. Remarkably, a meta-analysis of included studies revealed significant reduction of leptin levels in individuals on CoQ_10_ supplementation when compared to those on placebo (SMD: −0.59 [95% CI: −0.98, −0.21]; *I*^2^ = 0%, *p* = 0.37) (Figure 2B).

#### 2.4.2. CoQ_10_ Supplementation Ameliorated Pro-Inflammatory Markers

**Figure 3:**
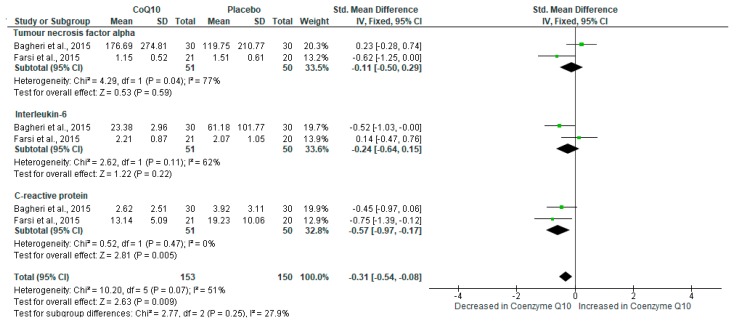
The impact of coenzyme Q_10_ (CoQ_10_) supplementation on markers of inflammation, such as tumor necrosis factor-alpha (TNF-α), interleukin-6 (IL-6) and C-reactive protein (CRP) in individuals with metabolic syndrome.

Consistent with the dysregulation in adipokine levels, metabolic syndrome has been associated an aggravation in inflammation [38,39], and this state that is characterized by increased pro-inflammatory cytokines and acute phase reactants such as CRP, IL-6, and tumor necrosis factor alpha (TNF-α) [13,14]. Interestingly, pooled estimates of inflammation markers showed a significant decrease in individuals on CoQ_10_ supplements when compared to controls placebo (SMD: −0.31 [95% CI: −0.54, −0.08]; *I*^2^ = 51%, *p* = 0.07) (Figure 3).

#### 2.4.3. CoQ_10_ Supplementation Reduced Markers of Lipid Peroxidation

**Figure 4:**
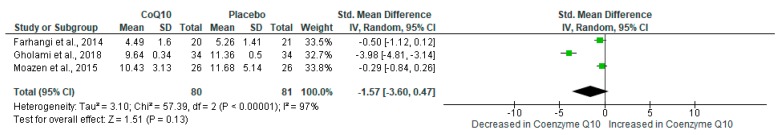
The impact of coenzyme Q_10_ (CoQ_10_) supplementation on malondialdehyde (MDA) levels as a maker of lipid peroxidation in individuals with metabolic syndrome.

It is well-recognized that individuals with metabolic syndrome have increased levels of oxidative stress, especially lipid peroxidation [41]. The latter is also a relevant target of CoQ_10_ in light of its critical role as a lipophilic antioxidant [41]. Interestingly, CoQ_10_ supplementation reduced MDA levels, as a marker of lipid peroxidation, when compared to placebo (SMD: −1.57 [95% CI: −3.60, 0.47]; *I*^2^ = 97%, *p* < 0.00001) (Figure 4).

#### 2.4.4. CoQ_10_ Supplementation Improved Blood Glucose Control

**Figure 5:**
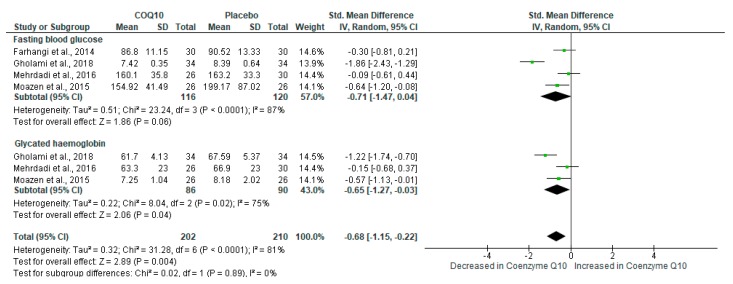
The impact of coenzyme Q_10_ (CoQ_10_) supplementation on basic metabolic markers such as fasting plasma glucose (FPG) levels and glycated hemoglobin (HbA1c).

Abnormally raised blood glucose levels are a prominent feature of T2D, consistent with the development of metabolic syndrome [4,42]. Interestingly, the results showed that CoQ_10_ supplementation significantly improved glucose control in individuals with metabolic syndrome (SMD: −0.68 [95% CI: −1.15, −0.22]; *I*^2^ = 81%, *p* < 0.00001) (Figure 5). Despite substantial levels of heterogeneity, the test for subgroup differences showed no significant subgroup effect (*p* = 0.89).

#### 2.4.5. CoQ_10_ Supplementation Did Not Impact Liver Function in Individuals with NAFLD

**Figure 6:**
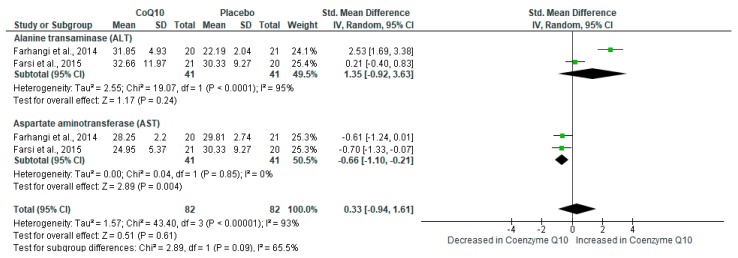
The effect of coenzyme Q_10_ (CoQ_10_) supplementation on liver function measured using alanine transaminase (ALT) and aspartate transaminase (AST) in individuals with non-alcoholic fatty liver disease (NAFLD).

Briefly, NAFLD remains one of the major characteristic features of metabolic syndrome [43], and this condition is persistent with the rise in hepatic enzymes such as alanine transaminase and aspartate transaminase (AST). Here, pooled estimates showed a small effect size in the liver function among individuals on CoQ_10_ supplements versus those on placebo (SMD: 0.33 [95% CI: −0.94, 1.61]; *I*^2^ = 93%, *p* < 0.00001) (Figure 6). Despite substantial levels of heterogeneity, the test for subgroup differences showed no significant subgroup effect (*p* = 0.09). The overall summary of findings on the beneficial effects of CoQ_10_ supplementation in individuals with metabolic syndrome is provided in Table 2.

## 3. Discussion

Emerging research supports the beneficial effects of CoQ_10_ against metabolic complications [32,34,35,44,45]. Several published RCTs suggest that CoQ_10_ can regulate adipokine levels, including those of leptin and adiponectin, to impact metabolic function. As a prime example, Gholami et al. 2018 [36] reported that CoQ_10_ supplementation in women with T2D was effective in increasing the concentrations of adiponectin or the adiponectin/leptin ratio, including reducing MDA levels which could translate to improved insulin sensitivity and amelioration of oxidative stress. Although Mehrdadi and colleagues [35] recorded a decline in adipolin levels with CoQ_10_ supplementation, this dietary compound could improve glucose homeostasis by reducing HbA1c concentrations. Perhaps suggesting that the anti-adipogenic effects of CoQ_10_ [46] are important in improving glucose homeostasis though not affecting adipolin in these patients. Like adiponectin, adipolin can impact the metabolic syndrome by attenuating atherosclerosis and improving insulin sensitivity, as reviewed elsewhere [47]. In another RCT [34], CoQ_10_ could lower lipid peroxidation by reducing MDA levels, albeit not significantly affecting FPG, HbA1c or adiponectin levels in patients with T2D. Apparently, dose selection could explain such limitation since diabetic individuals were treated with a dose of 100 mg CoQ_10_, which was much lower than the other two RCTs [35,36], which showed beneficial effects with 200 mg dose of ubiquinone on modulating glucose metabolism.

Nevertheless, the overall pooled estimates in the current study showed that CoQ_10_ supplementation significantly increased adiponectin levels in individuals with metabolic syndrome, which is concomitant to improved glucose control, and reduced levels of leptin and pro-inflammatory markers such as IL-6, CRP, and TNF-α. Apparently, circulation levels of adipokines such as leptin and adiponectin can act as biomarkers to evaluate obesity-associated complications, including low-grade inflammation [48]. While leptin is considered a crucial link between the compromised metabolic state and exacerbation of inflammation [40], enhanced adiponectin levels remain essential for effective regulation of glucose and lipid metabolism to improve metabolic function [38]. Interestingly, certain pharmacological interventions such as stragaloside II and isoastragaloside I have been found to selectively enhance adiponectin secretion in primary adipocytes without any apparent effects on other adipokines [49]. Thus, making therapeutic regulation of adipokine levels, including associated pro-inflammatory responses, as explored in the current study, an interesting strategy to protect against the metabolic syndrome.

Beyond controlling hyperglycemia and makers of lipid peroxidation or inflammation in those with T2D, other RCTs support the beneficial effects of CoQ_10_ in patients with NAFLD. Briefly, Farsi and colleagues [27] demonstrated that CoQ_10_ supplementation in patients with NAFLD limits liver damage by lowering the actions of AST and gamma-glutamyl transpeptidase, in connection with reducing the levels of CRP and TNF-α. Interestingly, such findings are consistent with elevated serum levels of adiponectin in patients with NAFLD [27]. Although these benefits could be observed, pooled estimates showed that CoQ_10_ did not show a significant effect in improving liver function in those with NAFLD. A possible reason for not observing significant effects with CoQ_10_ supplementation could be the limited number of RCTs included in the current meta-analysis.

Looking at the impact of other adipokine markers, Farhangi and colleagues [25] demonstrated that CoQ_10_ could significantly reduce serum levels of AST and MDA when compared to the placebo group. Here, changes in serum FPG were a significant predictor of fluctuations in serum adipokines such as vaspin, chemerin and pentraxin-3. Although not widely explored as adiponectin and leptin, adequate regulation of serum levels of other adipokines such as vaspin, chemerin, and pentraxin 3 has been associated with improved insulin sensitivity and vascular function [50,51,52]. Alternatively, Nesami and co-workers [26] found CoQ_10_ supplementation to be effective in decreasing some pro-inflammatory factors like IL-6 and CRP, in association with increasing adiponectin levels in patients with mild hypertension. Thus, broadly indicating that by effectively regulating adipokine levels, CoQ_10_ can greatly impact metabolic health in connection to ameliorating inflammation and oxidative stress in patients with T2D, NAFLD, or hypertension, as increasingly discussed [28,29,30,53]. Therapeutic benefits of CoQ_10_ supplementation are essential important since its deficiency in the body or enhanced oxidation status has been linked with compromised metabolic function [24,54,55]. In fact, Gholami and colleagues [36] demonstrated that at the end of the intervention, CoQ_10_ supplementation significantly improved its serum levels, including that of adiponectin while reducing that of MDA in patients with T2D. Thus, inferring that increased intake of CoQ_10_ could lead to its enhanced endogenous levels, which are vital for the amelioration of oxidative stress, especially lipid peroxidation products. Also highlighting the importance of understanding the link between CoQ_10_ intake and its delivery in vivo, as discussed elsewhere [56].

## 4. Materials and Methods

The current meta-analysis was prepared in accordance with the Preferred Reporting Items for Systematic reviews and Meta-Analysis (PRISMA) guidelines [57]. Subsequently, supplementary file S1 provides a PRISMA checklist for this meta-analysis.

### 4.1. Strategy to Search RCTs

The systematic search for eligible studies was conducted using electronic databases such as MEDLINE, Cochrane Library, and EMBASE from inception up to 29 February 2020 by two independent investigators (Phiwayinkosi V. Dludla, P.V.D.; and Tawanda M. Nyambuya, T.M.N.). In cases of disagreement, a third investigator (Bongani B. Nkambule, B.B.N.) was consulted for adjudication. The primary search was limited to RCTs, reporting the impact of CoQ_10_ supplementation on adipokine levels, glucose control, liver function, including makers of inflammation and oxidative stress in individuals with metabolic syndrome. Within the same cohort, individuals receiving placebo were used a comparative control. For an optimal search strategy, Medical Subject-Heading (MeSH) and text words such as “coenzyme Q_10_”, “metabolic syndrome”, “adipokine”, “inflammation”, and their matching synonyms and connected words or phrases were modified for each database used. In addition, a manual search was also performed through references of articles, abstracts, preprint platforms (to identify unpublished studies), which is essential to identify relevant evidence. There were no language restrictions applied in the search strategy, whilst EndNote version 10 (Clarivate Analytics, Philadelphia, USA) was used to manage the reference list and eliminate duplicates, as previously reported [58].

### 4.2. Inclusion and Exclusion Criteria

The current meta-analysis encompassed RCTs evaluating the impact of CoQ_10_ on adipokine levels in correlation with basic metabolic parameters in adults (>18 years) with metabolic syndrome. In brief, included RCTs were those that evaluating the use of CoQ_10_ as an intervention, contained the comparison group on placebo, and reported on adipokine levels in individuals with metabolic syndrome. Animal or in vitro studies were not included, while other exclusions included books, cohort or observational studies, letters, and case reports. Review articles were only scanned for RCTs. Furthermore, studies not reporting on the effects of CoQ_10_ on adipokine levels in patients without metabolic syndrome were excluded.

### 4.3. Data Extraction and Assessment of Quality

For data extraction, P.V.D. and T.M.N independently assessed all relevant articles and cautiously selected those that met the inclusion criteria. Any disagreements were resolved by referring a third investigator (B.B.N.). The primary outcome of the meta-analysis was to establish the impact of CoQ_10_ supplementation on adipokine levels in conditions of metabolic syndrome. Another significant objective was to understand whether the modulation of adipokine levels by CoQ_10_ supplementation related to improved inflammatory response and amelioration of oxidative stress markers. To achieve this, pertinent data items from each RCT included, such as name and year of publication, the country where the study was conducted, sample and gender distribution, in addition to CoQ_10_ dosage and intervention period were extracted. Moreover, the risk of bias was assessed by two investigators (V.M. and K.M.) using the modified Downs and Black checklist, which is suitable for both randomized and non-randomized studies [59,60]. Any differences were determined by referring the third investigator (T.M.N.). The same investigators were also tasked with evaluating the quality of evidence across the selected RCTs by using the Grading of Recommendations Assessment Development and Evaluation (GRADE) approach [61].

### 4.4. Statistical Analysis

Statistical analysis was performed using RevMan software (version 5.0; Cochrane Collaboration, Oxford, UK). Higgin’s *I^2^* statistics was used to test for statistical heterogeneity [62]. The random or fixed-effects models were used depending on the levels of statistical heterogeneity, as previously discussed [63]. Cohen’s *d* method was employed to interpret effect sizes, whereby a standardized mean difference of 0.2, 0.5. and 0.8 were regarded as small, medium, and large effect, respectively [64]. This method makes use of standardized mean difference (SMD) and not actual mean differences to measure effect sizes. Briefly, SMD is calculated as per the Cochrane guidelines, where it is recommended when estimating the effect size of pooled studies which report a similar outcome using a variety scales (SI units) [65]. While a *p*-value <0.05 was considered statistically significant. The inter-rater reliability was evaluated for both the encompassed studies and risk of bias by means of Cohen’s kappa. For example, a kappa value of <0.00 was considered as poor strength of agreement, 0.00–0.20 as minor agreement, 0.21–0.40 as fair agreement, 0.41–0.60 as reasonable agreement, 0.61–0.80 as significant agreement, and 0.81–1.00 as perfect agreement [33].

## 5. Study Limitations

The limitation of the current meta-analysis includes the low number of RCTs included, which significantly reduces the confidence in the level of the. reported findings, especially those on its beneficial properties against NAFLD. Thus, suggesting that additional RCTs are necessary to improve our understanding of the correlation between adipokine regulation and metabolic abnormalities in patients with NAFLD. Another limitation, most included RCTs did not detect serum levels of CoQ_10_ post-intervention, which is a crucial aspect to assess to determine its bioavailability and its therapeutic potential.

## 6. Conclusions

Coenzyme Q_10_ (CoQ_10_) is a dietary compound with established antioxidant properties and is increasingly investigated for its ameliorative effects against various metabolic complications. Evidence from other RCTs has been synthesized to inform on the impact of CoQ_10_ on inflammation, oxidative stress, or insulin resistance [28,29,30,66,67,68], including its modulation of adiponectin [31]. However, the current meta-analysis provides novel findings on the broad effects of this compound on the regulation of various adipokines on diverse metabolic complications. Indeed, summarized evidence suggests that optimal regulation of adipokine markers such as adiponectin and leptin is crucial for the beneficial effects of CoQ_10_ in attenuating oxidative stress and inflammation in conditions on metabolic syndrome.

## Figures and Tables

**Table 1:** Characteristic features of included studies and the reported impact of coenzyme Q_10_ (CoQ_10_) on adipokine function and inflammatory response.

Study	Study Size	Male, *n* (%)	Age (Years)	CoQ_10_ Dosage and Duration	Main Findings
**Farhangi et al. (2014)** [25]	41 Non-alcoholic fatty liver disease (NAFLD) patients	31 (74)	42.0 ± 10.8	CoQ_10_ at 100 mg for 4 weeks	CoQ_10_ significantly reduced waist circumference and serum aspartate transaminase (AST) and total antioxidant capacity (TAC) concentrations compared to the placebo-treated group. In the stepwise multivariate linear regression model, change in serum fasting serum glucose was a significant predictor of changes in serum vaspin, chemerin, and pentraxin 3
**Moazen et al. (2015)** [34]	52 type 2 diabetic (T2D) patients	28 (54)	51.7 ± 7.3	CoQ_10_ at 100 mg for 8 weeks	CoQ_10_ reduced malondialdehyde (MDA) levels; however, fasting blood glucose (FPG), glycated hemoglobin (HbA1c) and adiponectin levels showed no significant differences when compared to the placebo control
**Nesami et al. (2015)** [26]	60 patients suffering from mild hypertension	17 (28)	48.8 ± 5.9	CoQ_10_ at 100 mg for 12 weeks	CoQ_10_ was effective in decreasing some pro-inflammatory factors, such as IL-6 and C-reactive protein (CRP), and in increasing adiponectin levels
**Farsi et al. (2016)** [27]	41 NAFLD patients	28 (68)	Not reported	CoQ_10_ at 100 mg for 3 months	CoQ_10_ significantly reduced AST and gamma-glutamyl transpeptidase, CRP, tumor necrosis factor-alpha (TNF-α), and the grades of NAFLD. In addition, patients who received CoQ_10_ supplement had higher serum levels of adiponectin and considerable changes in serum leptin
**Mehrdadi et al. (2017)** [35]	56 patients with T2D	32 (57)	47.0 ± 8	CoQ_10_ at 200 mg/day for 12 weeks	CoQ_10_ reduced HbA1c, although interestingly, adipolin levels declined simultaneously. CoQ_10_ modulated glucose homeostasis, which was expected to be mediated by increasing adipolin. Similar mechanisms of action of CoQ_10_ and adipolin may justify lowering the effect of CoQ_10_ on adipolin. In addition, the possible anti-adipogenic effect of CoQ_10_ might explain the significant reduction in weight and waist circumference and hence the adipolin decrease
**Gholami et al. (2018)** [36]	68 patients with T2D	Not reported	48.8 ± 6.4	CoQ_10_ at 200 mg/d for 8 weeks	CoQ_10_ supplementation in women with T2D was effective in elevation of adiponectin and the adiponectin/leptin ratio (A/L), MDA and 8-isoprostane which could result in improving insulin resistance and modulating oxidative stress

**Table 2:** Summary of findings.

Coenzyme Q_10_ (CoQ_10_) Supplementation Compared to Placebo						
Patient or population: Adults (≥18 years of age) with Metabolic Syndrome Intervention: CoQ_10_ Supplementation Comparison: Individuals with Metabolic Syndrome Receiving Placebo						
Outcomes	Anticipated Absolute Effects * (95% CI)		Relative Effect (95% CI)	№ of Participants (Studies)	Certainty of the Evidence (GRADE)	Comments
	Risk with Placebo	Risk with CoQ_10_ Supplementation				
Adipokine control measured using adiponectin levels	-	The mean level in the intervention group was 1.44 higher (0.13 lower to 3.00 higher)	-	221 (4 RCT’s studies)	⨁⨁⨁⨁ HIGH	
Inflammation measured by C-reactive protein (CRP) levels	-	The mean level in the intervention group was 0.57 lower (0.97 lower to 0.17 lower)		101 (2 RCT’s studies)	⨁⨁⨁⨁ HIGH	
Oxidative stress measured by malondialdehyde (MDA) levels	-	The mean level in the intervention group was 1.57 lower (3.60 lower to 0.47 higher)		161 (3 RCT’s studies)	⨁⨁⨁⨁ HIGH	
Glucose control measured by glycated haemoglobin (Hb1Ac) levels	-	The mean level in the intervention group was 0.65 lower (1.27 lower to 0.03 lower)		176 (3 RCT’s studies)	⨁⨁⨁⨁ HIGH	
Liver function measured by aspartate transaminase (AST) levels	-	The mean level in the intervention group was 0.66 lower (1.10 lower to 0.21 lower)		82 (2 RCT’s studies)	⨁⨁⨁⨁ HIGH	

* The risk in the intervention group (and its 95% confidence interval) is based on the assumed risk in the comparison group and the relative effect of the intervention (and its 95% CI). CI: Confidence interval; MD: mean difference; OR: odds ratio; NE: not estimable GRADE Working Group grades of evidence. High certainty: We are very confident that the true effect lies close to that of the estimate of the effect. Moderate certainty: We are moderately confident in the effect estimate: The true effect is likely to be close to the estimate of the effect, but there is a possibility that it is substantially different. Low certainty: our confidence in the effect estimate is limited: the true effect may be substantially different from the estimate of the effect. Very low certainty: we have very little confidence in the effect estimate: The true effect is likely to be substantially different from the estimate of effect.

## Supplementary Materials

The following are available online at https://www.mdpi.com/1422-0067/21/9/3247/s1, Supplementary file S1: Preferred Reporting Items for Systematic reviews and Meta-Analysis (PRISMA) checklist.

## Abbreviations

ALT	Aspartate transaminase
CVD	Cardiovascular disease
CoQ_10_	Coenzyme Q_10_
CRP	C-reactive protein
FPG	Fasting plasma glucose
GRADE	Grading of Recommendations Assessment Development and Evaluation
HbA1c	Glycated hemoglobin
IL	Interleukin
MDA	Malondialdehyde
NAFLD	Non-alcoholic fatty liver disease
PRISMA	Preferred Reporting Items for Systematic reviews and Meta-Analysis
RCT	Randomized controlled trials
SMD	Standardized mean difference
T2D	Type 2 diabetes
TNF-α	Tumor necrosis factor alpha

## References

[1] Trikkalinou A., Papazafiropoulou A.K., Melidonis A. (2017). Type 2 diabetes and quality of life. World J. Diabetes.

[2] World Health Organization The Top 10 Causes of Death. https://www.who.int/news-room/fact-sheets/detail/the-top-10-causes-of-death.

[3] Pitsavos C., Panagiotakos D., Weinem M., Stefanadis C. (2006). Diet, exercise and the metabolic syndrome. Rev. Diabet. Stud..

[4] Grundy S.M. (2016). Overnutrition, ectopic lipid and the metabolic syndrome. J. Investig. Med..

[5] Saklayen M.G. (2018). The global epidemic of the metabolic syndrome. Curr. Hypertens. Rep..

[6] World Health Organization Noncommunicable Diseases Country Profiles 2014. https://www.who.int/nmh/publications/ncd-profiles-2014/en/.

[7] Afshin A., Forouzanfar M.H., Reitsma M.B., Sur P., Estep K., Lee A., Marczak L., Mokdad A.H., Moradi-Lakeh M., Naghavi M. (2017). Health effects of overweight and obesity in 195 countries over 25 years. N. Engl. J. Med..

[8] Heather L.C., Clarke K. (2011). Metabolism, hypoxia and the diabetic heart. J. Mol. Cell. Cardiol..

[9] Mosterd A., Hoes A.W. (2007). Clinical epidemiology of heart failure. Heart.

[10] Bril F., Cusi K. (2017). Management of nonalcoholic fatty liver disease in patients with type 2 diabetes: A call to action. Diabetes Care.

[11] Das H., Banik S. (2019). Prevalence of dyslipidemia among the diabetic patients in southern Bangladesh: A cross-sectional study. Diabetes Metab. Syndr..

[12] Schofield J.D., Liu Y., Rao-Balakrishna P., Malik R.A., Soran H. (2016). Diabetes dyslipidemia. Diabetes Ther..

[13] Waheed P., Naveed A.K., Farooq F. (2009). Levels of inflammatory markers and their correlation with dyslipidemia in diabetics. J. Coll. Physicians Surg. Pak..

[14] Aulinas A., Ramírez M.J., Barahona M.J., Valassi E., Resmini E., Mato E., Santos A., Crespo I., Bell O., Surrallés J. (2015). Dyslipidemia and chronic inflammation markers are correlated with telomere length shortening in Cushing’s syndrome. PLoS ONE.

[15] Rao V., Kiran R. (2011). Evaluation of correlation between oxidative stress and abnormal lipid profile in coronary artery disease. J. Cardiovasc. Dis. Res..

[16] Dludla P.V., Nkambule B.B., Jack B., Mkandla Z., Mutize T., Silvestri S., Orlando P., Tiano L., Louw J., Mazibuko-Mbeje S.E. (2018). Inflammation and oxidative stress in an obese state and the protective effects of gallic acid. Nutrients.

[17] Dludla P.V., Mazibuko-Mbeje S.E., Nyambuya T.M., Mxinwa V., Tiano L., Marcheggiani F., Cirilli I., Louw J., Nkambule B.B. (2019). The beneficial effects of N-acetyl cysteine (NAC) against obesity associated complications: A systematic review of pre-clinical studies. Pharmacol. Res..

[18] Calvano A., Izuora K., Oh E.C., Ebersole J.L., Lyons T.J., Basu A. (2019). Dietary berries, insulin resistance and type 2 diabetes: An overview of human feeding trials. Food Funct..

[19] Salehi B., Mishra A.P., Nigam M., Sener B., Kilic M., Sharifi-Rad M., Fokou P.V.T., Martins N., Sharifi-Rad J. (2018). Resveratrol: A double-edged sword in health benefits. Biomedicines.

[20] Qu H., Guo M., Chai H., Wang W.T., Gao Z.Y., Shi D.Z. (2018). Effects of coenzyme Q10 on statin-induced myopathy: An updated meta-analysis of randomized controlled trials. JAHA.

[21] Gutierrez-Mariscal F.M., Yubero-Serrano E.M., Villalba J.M., Lopez-Miranda J. (2019). Coenzyme Q(10): From bench to clinic in aging diseases, a translational review. Crit. Rev. Food Sci. Nutr..

[22] Shen Q., Pierce J.D. (2015). Supplementation of coenzyme Q10 among patients with type 2 diabetes mellitus. Healthcare.

[23] Littarru G.P., Tiano L. (2007). Bioenergetic and antioxidant properties of coenzyme Q10: Recent developments. Mol. Biotechnol..

[24] Orlando P., Chellan N., Louw J., Tiano L., Cirilli I., Dludla P., Joubert E., Muller C.J.F. (2019). Aspalathin-rich green rooibos extract lowers LDL-cholesterol and oxidative status in high-fat diet-induced diabetic vervet monkeys. Molecules.

[25] Farhangi M.A., Alipour B., Jafarvand E., Khoshbaten M. (2014). Oral coenzyme Q10 supplementation in patients with nonalcoholic fatty liver disease: Effects on serum vaspin, chemerin, pentraxin 3, insulin resistance and oxidative stress. Arch. Med. Res..

[26] Bagheri Nesami N., Mozaffari-Khosravi H., Najarzadeh A., Salehifar E. (2015). The effect of coenzyme Q10 supplementation on pro-inflammatory factors and adiponectin in mildly hypertensive patients: A randomized, double-blind, placebo-controlled trial. Int. J. Vitam. Nutr. Res..

[27] Farsi F., Mohammadshahi M., Alavinejad P., Rezazadeh A., Zarei M., Engali K.A. (2016). Functions of coenzyme Q10 supplementation on liver enzymes, markers of systemic inflammation, and adipokines in patients affected by nonalcoholic fatty liver disease: A double-blind, placebo-controlled, randomized clinical trial. J. Am. Coll. Nutr..

[28] Jorat M.V., Tabrizi R., Kolahdooz F., Akbari M., Salami M., Heydari S.T., Asemi Z. (2019). The effects of coenzyme Q10 supplementation on biomarkers of inflammation and oxidative stress in among coronary artery disease: A systematic review and meta-analysis of randomized controlled trials. Inflammopharmacology.

[29] Farsi F., Heshmati J., Keshtkar A., Irandoost P., Alamdari N.M., Akbari A., Janani L., Morshedzadeh N., Vafa M. (2019). Can coenzyme Q10 supplementation effectively reduce human tumor necrosis factor-α and interleukin-6 levels in chronic inflammatory diseases? A systematic review and meta-analysis of randomized controlled trials. Pharmacol. Res..

[30] Zhai J., Bo Y., Lu Y., Liu C., Zhang L. (2017). Effects of coenzyme Q10 on markers of inflammation: A systematic review and meta-analysis. PLoS ONE.

[31] Nazary-Vannani A., Ghaedi E., Salamat S., Sayyaf A., Varkaneh H.K., Mohammadi H., Djalali M. (2020). Effects of coenzyme Q10 supplementation on serum adiponectin levels: A systematic review and meta-analysis of randomized controlled trials. Curr. Drug ther..

[32] Gökbel H., Gergerlioğlu H.S., Okudan N., Gül I., Büyükbaş S., Belviranli M. (2010). Effects of coenzyme Q10 supplementation on plasma adiponectin, interleukin-6, and tumor necrosis factor-alpha levels in men. J. Med. Food.

[33] Landis J.R., Koch G.G. (1977). The measurement of observer agreement for categorical data. Biometrics.

[34] Moazen M., Mazloom Z., Ahmadi A., Dabbaghmanesh M., Roosta S. (2015). Effect of coenzyme Q10 on glycaemic control, oxidative stress and adiponectin in type 2 diabetes. J. Pak. Med. Assoc..

[35] Mehrdadi P., Kolahdouz Mohammadi R., Alipoor E., Eshraghian M.R., Esteghamati A., Hosseinzadeh-Attar M.J. (2017). The Effect of coenzyme Q10 supplementation on circulating levels of novel adipokine adipolin/CTRP12 in overweight and obese patients with type 2 diabetes. Exp. Clin. Endocrinol. Diabetes.

[36] Gholami M., Zarei P., Sadeghi Sedeh B., Rafiei F., Khosrowbeygi A. (2018). Effects of coenzyme Q10 supplementation on serum values of adiponectin, leptin, 8-isoprostane and malondialdehyde in women with type 2 diabetes. Gynecol. Endocrinol..

[37] Ouchi N., Parker J.L., Lugus J.J., Walsh K. (2011). Adipokines in inflammation and metabolic disease. Nat. Rev. Immunol..

[38] Achari A.E., Jain S.K. (2017). Adiponectin, a therapeutic target for obesity, diabetes, and endothelial dysfunction. Int. J. Mol. Sci..

[39] Ahima R.S., Osei S.Y. (2008). Adipokines in obesity. Front. Horm. Res..

[40] Iikuni N., Lam Q.L., Lu L., Matarese G., La Cava A. (2008). Leptin and Inflammation. Curr. Immunol. Rev..

[41] Bakhtiari A., Hajian-Tilaki K., Omidvar S., Nasiri Amiri F. (2017). Association of lipid peroxidation and antioxidant status with metabolic syndrome in Iranian healthy elderly women. Biomed. Rep..

[42] Al-Goblan A.S., Al-Alfi M.A., Khan M.Z. (2014). Mechanism linking diabetes mellitus and obesity. Diabetes Metab. Syndr. Obes..

[43] Paschos P., Paletas K. (2009). Non alcoholic fatty liver disease and metabolic syndrome. Hippokratia.

[44] Moradi M., Haghighatdoost F., Feizi A., Larijani B., Azadbakht L. (2016). Effect of coenzyme Q10 supplementation on diabetes biomarkers: A systematic review and meta-analysis of randomized controlled clinical trials. Arch. Iran Med..

[45] Dludla P.V., Nyambuya T.M., Orlando P., Silvestri S., Mxinwa V., Mokgalaboni K., Nkambule B.B., Louw J., Muller C.J.F., Tiano L. (2020). The impact of coenzyme Q10 on metabolic and cardiovascular disease profiles in diabetic patients: A systematic review and meta-analysis of randomized controlled trials. Endocrinol. Diab. Metab..

[46] Xu Z., Huo J., Ding X., Yang M., Li L., Dai J., Hosoe K., Kubo H., Mori M., Higuchi K. (2017). Coenzyme Q10 improves lipid metabolism and ameliorates obesity by regulating CaMKII-mediated PDE4 inhibition. Sci. Rep..

[47] Sargolzaei J., Chamani E., Kazemi T., Fallah S., Soori H. (2018). The role of adiponectin and adipolin as anti-inflammatory adipokines in the formation of macrophage foam cells and their association with cardiovascular diseases. Clin. Biochem..

[48] Inadera H. (2008). The usefulness of circulating adipokine levels for the assessment of obesity-related health problems. Int. J. Med. Sci..

[49] Xu A., Wang H., Hoo R.L., Sweeney G., Vanhoutte P.M., Wang Y., Wu D., Chu W., Qin G., Lam K.S. (2009). Selective elevation of adiponectin production by the natural compounds derived from a medicinal herb alleviates insulin resistance and glucose intolerance in obese mice. Endocrinology.

[50] Wada J. (2008). Vaspin: A novel serpin with insulin-sensitizing effects. Expert Opin. Investig. Drugs.

[51] Peri G., Introna M., Corradi D., Iacuitti G., Signorini S., Avanzini F., Pizzetti F., Maggioni A.P., Moccetti T., Metra M. (2000). PTX3, A prototypical long pentraxin, is an early indicator of acute myocardial infarction in humans. Circulation.

[52] Zanetti M., Bosutti A., Ferreira C., Vinci P., Biolo G., Fonda M., Valente M., Cattin L., Guarnieri G., Barazzoni R. (2009). Circulating pentraxin 3 levels are higher in metabolic syndrome with subclinical atherosclerosis: Evidence for association with atherogenic lipid profile. Clin. Exp. Med..

[53] Rosenfeldt F.L., Haas S.J., Krum H., Hadj A., Ng K., Leong J.Y., Watts G.F. (2007). Coenzyme Q10 in the treatment of hypertension: A meta-analysis of the clinical trials. J. Hum. Hypertens..

[54] Quinzii C.M., DiMauro S., Hirano M. (2007). Human coenzyme Q10 deficiency. Neurochem. Res..

[55] Rahman S., Clarke C.F., Hirano M. (2012). 176th ENMC International Workshop: Diagnosis and treatment of coenzyme Q(1)(0) deficiency. Neuromuscul. Disord..

[56] Zaki N.M. (2016). Strategies for oral delivery and mitochondrial targeting of CoQ10. Drug Deliv..

[57] Shamseer L., Moher D., Clarke M., Ghersi D., Liberati A., Petticrew M., Shekelle P., Stewart L.A. (2015). Preferred reporting items for systematic review and meta-analysis protocols (PRISMA-P) 2015: Elaboration and explanation. BMJ.

[58] Mahlangu T., Dludla P.V., Nyambuya T.M., Mxinwa V., Mazibuko-Mbeje S.E., Cirilli I., Marcheggiani F., Tiano L., Louw J., Nkambule B.B. (2019). A systematic review on the functional role of Th1/Th2 cytokines in type 2 diabetes and related metabolic complications. Cytokine.

[59] Downs S.H., Black N. (1998). The feasibility of creating a checklist for the assessment of the methodological quality both of randomised and non-randomised studies of health care interventions. J. Epidemiol. Community Health.

[60] O’Connor S.R., Tully M.A., Ryan B., Bradley J.M., Baxter G.D., McDonough S.M. (2015). Failure of a numerical quality assessment scale to identify potential risk of bias in a systematic review: A comparison study. BMC Res. Notes.

[61] Balshem H., Helfand M., Schünemann H.J., Oxman A.D., Kunz R., Brozek J., Vist G.E., Falck-Ytter Y., Meerpohl J., Norris S. (2011). GRADE guidelines: 3. Rating the quality of evidence. J. Clin. Epidemiol..

[62] Higgins J.P., Thompson S.G. (2002). Quantifying heterogeneity in a meta-analysis. Stat. Med..

[63] Schroll J.B., Moustgaard R., Gotzsche P.C. (2011). Dealing with substantial heterogeneity in Cochrane reviews. Cross-sectional study. BMC Med. Res. Methodol..

[64] Sullivan G.M., Feinn R. (2012). Using effect size-or why the p value is not enough. J. Grad. Med. Educ..

[65] Higgins J.P.T., Wells G.A. (2011). Cochrane Handbook for Systematic Reviews of Interventions.

[66] Mazidi M., Kengne A.P., Banach M., Lipid, Blood Pressure Meta-analysis Collaboration, G (2018). Effects of coenzyme Q10 supplementation on plasma C-reactive protein concentrations: A systematic review and meta-analysis of randomized controlled trials. Pharmacol. Res..

[67] Sangsefidi Z.S., Yaghoubi F., Hajiahmadi S., Hosseinzadeh M. (2020). The effect of coenzyme Q10 supplementation on oxidative stress: A systematic review and meta-analysis of randomized controlled clinical trials. Food Sci. Nutr..

[68] Huang H., Chi H., Liao D., Zou Y. (2018). Effects of coenzyme Q10 on cardiovascular and metabolic biomarkers in overweight and obese patients with type 2 diabetes mellitus: A pooled analysis. Diabetes Metab. Syndr. Obes..

