# Supplementary material for: Coenzyme Q10 Supplementation Improves Adipokine Levels and Alleviates Inflammation and Lipid Peroxidation in Conditions of Metabolic Syndrome: A Meta-Analysis of Randomized Controlled Trials
Source: Int J Mol Sci. 2020 May 4;21(9):3247. doi: 10.3390/ijms21093247 (PMC7247332; doi:10.3390/ijms21093247)
Supplement: Supplementary file 1 [file ijms-21-03247-s001.pdf]

PRISMA 2009 Checklist (Adapted for KIN 4400)

Section/topic	#	Checklist item	Reported on page #
**TITLE**			
Title	1	Identify the report as a literature review.	1
**ABSTRACT**			
Structured summary	2	Provide a structured summary including, as applicable: background; objectives; data sources; study eligibility criteria, participants, and interventions; study appraisal and synthesis methods; results; limitations; conclusions and implications of key findings;	1
**INTRODUCTION**			
Rationale	3	Describe the rationale for the review in the context of what is already known about your topic.	2
Objectives	4	Provide an explicit statement of questions being addressed with reference to participants, interventions, comparisons, outcomes, and study design (PICOS).	10
**METHODS**			
Eligibility criteria	5	Specify study characteristics (e.g., PICOS, length of follow-up) and report characteristics (e.g., years considered, language, publication status) used as criteria for eligibility, giving rationale.	10
Information sources	6	Describe all information sources (e.g., databases with dates of coverage) in the search and date last searched.	10
Search	7	Present full electronic search strategy for at least one database, including any limits used, such that it could be repeated.	10
Study selection	8	State the process for selecting studies (i.e., screening, eligibility).	10
Risk of bias in individual studies	9	Describe methods used for assessing risk of bias of individual studies (including specification of whether this was done at the study or outcome level).	10
Risk of bias across studies	10	Specify any assessment of risk of bias that may affect the cumulative evidence (e.g., publication bias, selective reporting within studies).	10
**RESULTS**			
Study selection	11	Give numbers of studies screened, assessed for eligibility, and included in the review, with reasons for exclusions at each stage, ideally with a flow diagram.	2-3

PRISMA 2009 Checklist (Adapted for KIN 4400)

Section/topic	#	Checklist item	Reported on page #
Study characteristics	12	For each study, present characteristics for which data were extracted (e.g., study size, PICOS, follow-up period) and provide the citations.	4
Synthesis of results of individual studies	13	For all outcomes considered (benefits or harms), present, for each study: (a) summary of results and (b) relationship to other studies under review (e.g. agreements or disagreements in methods, sampling, data collection or findings).	5-8
**DISCUSSION**			
Summary of evidence	14	Summarize the main findings including the strength of evidence for each main outcome; consider their relevance to key groups (e.g., healthcare providers, users, and policy makers).	9-10
Limitations	15	Discuss limitations at study and outcome level (e.g., risk of bias), and at review-level (e.g., incomplete retrieval of identified research, reporting bias).	11
**CONCLUSION**			
Conclusions	16	Provide a general interpretation of the results in the context of other evidence, and implications for future research.	11

Adapted from: Moher D, Liberati A, Tetzlaff J, Altman DG, The PRISMA Group (2009). Preferred Reporting Items for Systematic Reviews and Meta-Analyses: The PRISMA statement. *PLoS Medicine*, 6(6), e1000097. doi:10.1371/journal.pmed1000097

For more information, visit: **www.prisma-statement.org**.
